# Microbial industrial enzymes: An annotated selection of World Wide Web sites relevant to the topics in microbial biotechnology

**DOI:** 10.1111/1751-7915.13389

**Published:** 2019-02-25

**Authors:** Lawrence P. Wackett

**Affiliations:** ^1^ Department of Biochemistry, Molecular Biology and Biophysics BioTechnology Institute University of Minnesota St. Paul MN 55108 USA

## Microbial enzymes used in industry

https://www.sciencedirect.com/science/article/pii/B978012803725600011X

This review article provides a good rundown of the major industrial enzyme types and their specific applications.

## Microbial industrial enzymes

https://www.ncbi.nlm.nih.gov/pmc/articles/PMC4991975/

This review addresses commercial enzymes from the standpoint of the specific industries that enzymes serve currently and it includes some prospects for future applications.

## Microbial enzyme applications for food

https://www.ncbi.nlm.nih.gov/pmc/articles/PMC5956270/

This review focuses on enzymes in the food industries. It points out that microbes are the preferred source for these enzymes due to a lower cost and more consistent means of production.

## Biotechnology of microbial enzymes

https://www.elsevier.com/books/biotechnology-of-microbial-enzymes/brahmachari/978-0-12-803725-6

This is the website for a book dealing with the production and industrial applications of microbial enzymes.

## GRAS process for microbial enzymes

https://www.liebertpub.com/doi/full/10.1089/ind.2016.0011

GRAS stands for Generally Regarded As Safe. It is the standard for regulatory approval of substances expected to become an additive to a food product, including enzymes. To be be considered safe, food enzymes are typically produced in host organisms, that are deemed to be GRAS, such as *Bacillus subtilis*.

## Pectic enzymes in food and wine industries

https://www.intechopen.com/books/food-industrial-processes-methods-and-equipment/microbial-pectic-enzymes-in-the-food-and-wine-industry

Pectic enzymes are widely used in the food industry, largely dealing with beverages derived from fruits and vegetables.

## Metagenomics for mining new applied microbial enzymes

https://www.scitechnol.com/peer-review/metagenomics-prospective-in-biomining-the-microbial-enzymes-f8DZ.php?article_id=7172

For many enzyme applications, there is interest in discovering new enzymes with properties preferred over the existing enzymes. Metagenomic sequencing is a source for finding new sequences and enzymes.

## DuPont industrial biosciences

http://biosciences.dupont.com

The DuPont industrial biosciences site deals with all bioscience products. Enzymes are a major component, and DuPont is one of the world's largest enzyme producers.

## Enzymes at work: Novozymes

https://www.novozymes.com/-/Project/Novozymes/.../Enzymes_at_work.pdf

This web pamphlet is a little dated but provides an excellent overview of many commercial enzymes and their specific applications in industry.

## BASF industrial enzyme solutions

https://www.basf.com/global/en/products/cross-industry-solutions/enzymes.html

The large chemical company BASF also has a major enzyme division, and this website highlights their activities.

## Codexis enzyme engineering

https://www.codexis.com

Codexis focuses on enzyme modifications for industrial purposes. In many cases, especially for pharmaceutical applications, enzyme reactivity toward a specific synthetic intermediate needs to be improved to become cost‐effective.

## Biocatalysis at DSM

https://www.bio.org/sites/default/files/David%20Ager.pdf

DSM is a large traditional chemical company that has developed extensive proficiency with enzyme‐based catalysis.

## Amano enzymes

https://www.amano-enzyme.com

Amano is a specialty enzyme company that started in Japan in 1899 as a pharmaceutical company.

## Enzyme regulatory compliance

https://www.crunchbase.com/organization/enzyme-2#section-overview

Like all biotech products, they must meet regulatory compliance to go to the market. A new industry is emerging to help other companies rapidly and efficiently meet regulatory compliance.

## Global enzymes market

https://www.businesswire.com/news/home/20180628006408/en/Global-Enzymes-Market-Report-2018-Analysis-Type

This website is an entry to market information on the enzyme industry and prospects for growth.

## Industrial enzymes market

https://www.mordorintelligence.com/industry-reports/global-industrial-enzymes-market-industry

This website is on current and future projected market information on the enzyme industry.

## Subtilisin: Protein DataBank

http://www.rcsb.org/pdb/results/results.do?tabtoshow=Current&qrid=1C55793E

These webpages link to X‐ray structure information on a major industrial protease. There have been many modifications of this enzyme and there are 297 structures available for the search on ‘subtilisin.’

## Efficacy of commercial phytases

https://www.aspergillus.org.uk/content/efficacy-different-commercial-phytase-sources-and-development-phosphorus-release-curve

Phytases are used in animal nutrition to release phosphate from phytic acid.

## Glucose isomerase

https://www.slideshare.net/reshvenkatesh/glucose-isomerase

This slideshare provides information on glucose isomerase. Glucose isomerase in an important industrial enzyme, typically used in immobilized form to produce high‐fructose corn syrup.

